# Racial and Socioeconomic Differences in Distance Traveled for Elective Hip Arthroplasty

**DOI:** 10.5435/JAAOSGlobal-D-22-00021

**Published:** 2022-04-05

**Authors:** Matthew Orringer, Heather Roberts, Derek Ward

**Affiliations:** From the University of California, San Francisco School of Medicine, San Francisco, CA (Mr. Orringer), and the Department of Orthopaedic Surgery (Dr. Roberts and Dr. Ward), University of California, San Francisco.

## Abstract

**Introduction:**

There are data that disparities exist in access to total hip arthroplasty (THA). However, to date, no study has examined the relationship between distance traveled to undergo THA and patient demographic characteristics, such as race, insurance provider, and income level as well as postoperative outcomes.

**Methods:**

Data from the Healthcare Cost and Utilization Project, American Hospital Association, and the United States Postal Service were used to calculate the geographic distance between 211,806 patients' population-weighted zip code centroid points to the coordinates of the hospitals at which they underwent THA. We then used Healthcare Cost and Utilization Project data to examine the relationships between travel distance and both patient demographic indicators and postoperative outcomes after THA.

**Results:**

White patients traveled farther on average to undergo THA as compared with their non-White counterparts (17.38 vs 13.05 miles) (*P* < 0.0001). Patients with commercial insurance (17.19 miles) and Medicare (16.65 miles) traveled farther on average to receive care than did patients with Medicaid insurance coverage (14.00 miles) (*P* = 0.0001). Patients residing in zip codes in the top income quartile traveled farther to receive care (18.73 miles) as compared with those in the lowest income quartile (15.31 miles) (*P* < 0.0001). No clinically significant association was found between travel distance and adverse postoperative outcomes after THA.

**Discussion:**

Race, insurance provider, and zip code income quartile are associated with differences in the distance traveled to undergo THA. These findings may be indicative of underlying disparities in access to care across patient populations.

Total hip arthroplasty (THA) is a highly successful and frequently conducted operation.^1,2^ Prior research has demonstrated disparities in access to care and outcomes after joint arthroplasty operations.^3-5^ Singh et al^3^ found that African American patients had longer average length of stay after both THA and total knee arthroplasty (TKA), while White patients had higher patient-reported outcome measure scores. Another study found that White patients, those with commercial insurance coverage, and those earning greater than $25,000 per year had a shorter average length of stay.^4^ In addition, a review of the literature found that Black and Hispanic patients are less likely to undergo total joint arthroplasty operations as compared with White patients and have less favorable outcomes postoperatively. The review similarly found that patients with high socioeconomic status (SES) are more likely to undergo total joint arthroplasty and had better outcomes after the procedure.^5^

There are data from other surgical fields that have shown differences in travel distance based on patient demographics. For example, one study regarding the initial treatment of breast cancer showed that patients who traveled farther were younger, more likely to be White, more likely to have private insurance, and with fewer medical comorbidities.^6^ However, although there are known disparities in access to total joint arthroplasty and postoperative outcomes, there are limited data on whether these disparities exist in how far patients travel to receive care. One study found that patients with more severe indications for TKA were more likely to travel farther to receive their operations.^7^ Another study found that travel distance was not associated with increased length of stay after TKA.^8^ Beck et al^9^ similarly found no differences in adverse outcomes after surgery for femoroacetabular impingement syndrome based on the distance traveled to receive care. In addition, a number of previous studies have examined the relationship between patient demographics and postoperative outcomes of orthopaedic procedures.^8,10,11^ However, these studies did not analyze disparities for travel distance.

The goal of this study was to evaluate the relationship between distance traveled to receive elective THA and patient demographic characteristics and to analyze the relationship between travel distance and postoperative adverse outcomes after THA. We hypothesized that older patients, those with higher Charlson Comorbidity Index (CCI) scores, and individuals residing in zip codes with lower median income levels would be associated with increased travel distance. Furthermore, we hypothesized that the travel distance required for patients to receive specialized care might be a marker of comparatively poor access to healthcare services in general and therefore may predict relatively worse surgical outcomes. In addition, patients with comorbid conditions may be referred to tertiary care centers, and thus travel farther, to undergo THA with appropriate postoperative care.

## Methods

### Data Collection

We used the Healthcare Cost and Utilization Project State Inpatient Databases from Florida and New York to identify patients who underwent THA from 2006 to 2014. Patient demographics and comorbidities and data on postoperative adverse events were acquired (see Appendix A, http://links.lww.com/JG9/A200). We used the American Hospital Association database to collect the latitude and longitude coordinates for the hospitals represented in this study. The UnitedStatesZipCodes.org Enterprise database was used to obtain population-weighted zip code centroids for the patients in this study.^12^ This provided latitude and longitude coordinate points that could be used to closely approximate the distance between the patients' residences and the hospitals at which they received care.

### Exclusion Criteria

We excluded patients younger than 18 years, those who underwent simultaneous bilateral surgery, and those who underwent emergency surgery. Patients with missing zip code or hospital data were also excluded.

### Data Analysis

To calculate the distance traveled to receive care, we used the “geodist” command in Stata, which computes distance using the great circle equation. The great circle equation computes the shortest distance between two points on a sphere as measured by the distance traveled along the surface of the sphere. Because the Earth is nearly a perfect sphere, this equation is accurate within approximately 0.5%.^13^

All statistical analyses were conducted using Stata MP 16 analytical software (StataCorp, LLC). We analyzed whether travel distance was associated with differences in patient demographic data including preoperative comorbidities, zip code median income quartile, race, age, and insurance provider. Linear regression analysis was conducted to identify associations between distance traveled and continuous variables, specifically age and CCI. One-way analyses of variance were used for travel distance and categorical demographic variables: race, insurance provider, and zip code median income quartile. Using logistic regression, we evaluated whether the distance traveled to receive care was associated with differences in postoperative outcomes. Statistical significance was set at *P* < 0.05.

## Results

In New York and Florida, 262,912 patients received THA operations between 2006 and 2014. Of these, we excluded 25,487 for undergoing emergent procedures, 25,467 for missing location data, and 152 for being younger than 18 years. Thus, 211,806 patients were included in our analysis. Our cohort of patients was 55.9% female, with an average age of 66.25 years (SD: 12.06 years), and had an average CCI of 2.89 (SD: 1.74). Most of the patients were White (85.6%) and had Medicare insurance coverage (59.2%). Each of the four zip code median income quartile groups was well-represented with the most patients in the second lowest bracket (29.5%) and the fewest patients in the top income group (21.7%). See Table 1 for summary of these data.

**Table 1:** Demographic Data

Variable	
Age, yrs, mean ± SD	66.25 ± 12.06
Charlson Comorbidity Index, mean ± SD	2.89 ± 1.74
Sex, n (%)	
Male	93,393 (44.1)
Female	118,413 (55.9)
Race, n (%)	
Asian	749 (0.4)
Black	13,284 (6.3)
Hispanic	9,577 (4.5)
Native American	359 (0.1)
White	181,327 (85.6)
Missing or others	6,510 (3.1)
Insurance provider, n (%)	
Commercial	72,586 (34.3)
Medicaid	6,314 (3.0)
Medicare	125,496 (59.2)
Others	7,410 (3.5)
Hospital state, n (%)	
Florida	143,235 (67.6)
New York	68,571 (32.4)
Zip code income quartile, n (%)	
1 (lowest)	46,417 (21.9)
2	62,406 (29.5)
3	54,192 (25.6)
4 (highest)	45,991 (21.7)
Null	2,800 (1.3)

Older age and increased CCI were associated with decreased travel distance (*P* < 0.0001). However, for each of these analyses, the *R*^2^ value was less than 0.001 (Table 2). White patients traveled the farthest on average to undergo THA (17.38 miles). This was farther than that distance traveled by Black patients (10.94 miles), Hispanic patients (12.56 miles), Asian patients (12.53 miles), and Native American patients (15.98 miles). The relationship between race and distance traveled to receive care was statistically significant (*P* < 0.0001) (Table 3).

**Table 2:** Travel Distance Versus Continuous Demographic Variables

Variable	Coefficient	Confidence Interval	*R* ^2^	*P* Value
Age	−0.044	−0.066 to −0.022	0.0001	<0.001
Charlson Comorbidity Index	−0.407	−0.560 to −0.253	0.0001	<0.001

**Table 3:** Travel Distance Versus Categorical Demographic Variables

Variable	Observations	Mean	95% CI
Race			
White	177,003	17.38 miles	17.08-17.68
Black	13,284	10.94	10.43-11.45
Hispanic	9,577	12.56	11.68-13.45
Asian	749	12.53	9.58-15.48
Native American	359	15.98	9.62-22.35
Insurance provider			
Commercial	71,042	17.19 miles	16.80-17.58
Medicaid	6,172	14.00	13.37-14.63
Medicare	122,573	16.65	16.26-17.03
Self-pay	1,167	18.62	14.81-22.43
Zip code income quartile			
1 (lowest income)	45,935	15.31 miles	14.89-15.74
2	61,495	16.31	15.85-16.76
3	53,032	16.53	15.99-17.08
4 (highest income)	45,610	18.73	18.02-19.43

Patients with commercial insurance plans traveled 17.19 miles on average to undergo THA, compared with 14.00 miles for Medicaid patients and 16.65 miles for Medicare patients (*P* = 0.0001) (Table 3). Patients who used a self-pay method traveled an average of 18.62 miles, although the sample size for this group was small. Patients living in zip codes with higher median income levels traveled farther on average to receive care, with patients in the top quartile traveling 18.73 miles compared with patients in the lowest quartile traveling 15.31 miles (*P* < 0.0001) (Table 3).

Minimal association was observed between distance traveled to receive care and postoperative adverse outcomes. No outcome variables were noted to have correlation coefficients greater than 0.0004 (Table 4). Patients who traveled farther to undergo THA were less likely to have a mechanical malfunction of the hip within 365 days (*P* = 0.028, *R*^2^ = 0.0001), and patients who traveled farther were less likely to have genitourinary complications postoperatively (*P* = 0.002, *R*^2^ = 0.0003).

**Table 4:** Travel Distance Versus Postoperative Adverse Outcomes

Outcome	Odds Ratio	Confidence Interval	Correlation Coefficient (*R*^2^)	*P* Value
Stroke	1.000	0.999-1.001	0.0000	0.668
Death within 30 d	1.001	1.000-1.002	0.0004	0.069
Death within 90 d	1.000	0.999-1.001	0.0001	0.477
Death within 365 d	1.000	0.999-1.001	0.0000	0.514
Cardiac complication within 30 d	1.000	0.999-1.000	0.0000	0.435
DVT within 60 d	0.999	0.999-1.000	0.0001	0.099
Genitourinary complication within 30 d	0.999	0.998-0.9995	0.0003	0.002
Hematoma within 30 d	1.000	0.999-1.001	0.0000	0.822
Mechanical malfunction within 365 d	0.9995	0.999-0.9999	0.0001	0.028
Pulmonary embolism within 60 d	1.000	0.999-1.001	0.0000	0.995
Prosthetic joint infection within 365 d	0.999	0.998-1.000	0.0000	0.801
Respiratory complication within 30 d	0.999	0.998-1.000	0.0002	0.079
Readmission within 30 d	1.000	1.000-1.000	0.0000	0.875

DVT = deep vein thrombosis

## Discussion

This database study demonstrates that there are notable associations between distance traveled to receive elective THA and race, insurance, and zip code median income. Although the absolute differences in travel distance are modest, given the frequency with which THA is conducted, these data likely indicate meaningful disparities in access to surgical treatment. This may be especially true in urban areas in which the time it takes to drive one mile is much greater than that in a rural setting. Clinically significant differences in postoperative adverse outcomes were not observed based on travel distance. In addition, age and CCI were weakly associated with differences in distance traveled to receive care. This study is one of the first to specifically examine travel distance as a main comparative variable in this patient population.

Although the relationships between travel distance and both age and CCI are statistically significant, the correlation coefficients were small, indicating that the variance in travel distance is minimally explained by either of these variables. This is perhaps most surprising in the case of CCI because we hypothesized that patients with increased comorbidities would travel farther to receive more specialized care. Although one would imagine that those with increased comorbid conditions would require travel of a greater distance to tertiary centers, this was not borne out in our data. This is consistent with evidence that patients with fewer medical comorbidities traveled farther to receive breast cancer operations.^6^ It is possible that there are interactions between medical comorbidities, travel distance, and other factors, such as SES, which led to these results. For example, a patient with increased medical comorbidities but low SES may not be able to travel far to receive an operation even if they are referred to a distant site. This may lessen the overall effect of referral patterns and CCI scores on travel distance. Future research should more closely evaluate the relationship between medical comorbidities, travel distance, and SES.

The relationship between travel distance and race is a particularly interesting finding. We found that White patients traveled farther on average than Black, Hispanic, Asian, and Native American patients. Specifically, we found that White patients traveled 1.59 times farther than Black patients and 1.38 times farther than Hispanic patients. Given that THA is such a frequently conducted operation, this likely represents meaningful differences in access to care for many patients. These data may reflect that White Americans are more likely to live in rural settings, which are more geographically isolated and spread out over larger areas.^14^ It is also possible that White Americans have greater financial resources, on average, which allow them to travel farther to a hospital or surgeon of their choice.^15^ As such, future research should investigate the roles that urbanicity and income levels play in the relationship between race and distance traveled to receive care.

The association of insurance status and travel distance likely reflects economic resources. The difference between patients with commercial insurance and Medicare is small, likely reflecting the transition that many make from commercial insurance to Medicare once eligible at age 65 years. We found that Medicaid patients sought care closer to home than either of Medicare or commercially insured patients. It is possible that patients with Medicaid have more limited resources to seek out particular hospitals in which to receive care and thus are more likely to undergo THA at a nearby accepting hospital compared with patients with commercial insurance or Medicare. Although one might hypothesize that Medicaid recipients are more likely to live in urban centers with access to hospitals that are nearby geographically, there is evidence that among those younger than 65 years, Medicaid coverage is actually higher in rural areas (24%) than urban areas (22%).^16^ As such, it is likely that urbanicity is not the key factor in creating this disparity, but rather patient income levels and the ability to seek out a hospital farther away if desired.

We found that patients living in zip codes with higher median income levels traveled farther on average to receive care than those living in areas with lower average income levels. This suggests that individuals in higher income areas may be more likely to travel farther to receive specialized care or to seek out a particular hospital or surgeon as compared with individuals from lower income areas. It is possible that this is in part due to higher income individuals living in more suburban areas in which they may travel more miles to receive care, although it may not necessarily require more time to travel to their hospital of choice as compared with individuals from an urban center. These data are consistent with our finding that patients with Medicare and commercial insurance travel farther to receive care as compared with Medicaid recipients who generally have lower incomes. According to the US Census Bureau, White individuals have a higher median household income than both Hispanic and Black Americans, so it is possible that income levels also help to explain the differences between racial groups for mean travel distance to undergo THA.^15^

The relationship between distance traveled to receive care and postoperative adverse outcomes is also revealing. No outcome variables were noted to have *R*^2^ values greater than 0.0001, indicating that travel distance is minimally associated with any of these variables. We found that patients who traveled farther to undergo THA were less likely to have a mechanical malfunction of the hip within 365 days (*P* = 0.028) and that patients who traveled farther were less likely to have genitourinary complications postoperatively (*P* = 0.002). However, these statistically significant values are unlikely to be clinically relevant, given the small correlation coefficients and odds ratios. These data are consistent with previous findings that postoperative adverse outcomes did not vary based on travel distance to undergo surgery for femoroacetabular impingement syndrome.^9^ Although these studies are not directly comparable for the complications and degree of surgery, this suggests that other factors, perhaps including age, comorbid conditions, and severity of disease presentation, are more responsible for differences in postoperative outcomes than is the distance traveled to undergo THA. Some patients are undoubtedly traveling farther to receive more highly specialized care. However, the lack of adverse outcomes among otherwise healthy patients who *choose* to do so based on financial resources that provide them greater flexibility may “cancel out” the negative outcomes in those who think they *need* to travel or are referred to a tertiary care center based on their comorbidities or other factors. For this reason, additional research regarding surgical outcomes and patient demographics including the distances they have traveled to receive care is warranted to better understand and allocate medical resources to maximize equity in medical care.

Limitations of this study include the inherent disadvantages of large databases for coding accuracy and the inability to associate individual patient socioeconomic levels with variables of interest. In addition, there are likely interactions between patient demographic characteristics that may be interrelated, such as race and SES, as previously discussed. Examining the interplay between these variables may help to answer remaining questions regarding disparities in access to THA. Similarly, patient travel distance may also be influenced by urbanicity. As such, future studies may seek to evaluate the role that urbanicity plays in travel distance and access to care. In addition, although this study was able to analyze adverse outcomes, we were unable to investigate patient satisfaction and patient-reported outcomes. In addition, the use of population-weighted zip code centroids to calculate distance, while a relatively precise proxy measure, has drawbacks. Specifically, in large zip codes and those with fewer people, the calculated centroid points may be less accurate.^17^ Furthermore, the distance measures in this study are geographic, straight-line calculations between two coordinates, which is only an approximation for the true distance traveled by patients. Finally, although travel distance may be a marker of disparities in access to health care, there are likely additional factors that influence patient access to care as well, such as limitations set by insurance companies on where individuals can seek medical treatment. As such, future work may better address the patient-specific circumstances that influence where individuals ultimately undergo operations such as THA.

## Conclusions

This study provides evidence that there are notable differences in the distance traveled to receive elective THA among patients in different racial groups, patients with various health insurance providers, and patients of differing SES. Taken together, these results may have important implications for the relationship between income inequality and access to specialized health care. Future research may elucidate whether such findings are affected by factors such as urbanicity or the possibility of individuals from wealthier communities choosing to undergo THA at a specific hospital, even if it means traveling farther, because of their greater financial resources. These studies may help inform decision-making for medical resource allocation to increase healthcare equity.
